# Efficacy and Feasibility of Pain management and Patient Education for Physical Activity in Intermittent claudication (PrEPAID): protocol for a randomised controlled trial

**DOI:** 10.1186/s13063-019-3307-6

**Published:** 2019-04-16

**Authors:** Ukachukwu O. Abaraogu, Philippa M. Dall, Julie Brittenden, Wesley Stuart, Garry A. Tew, Jon Godwin, Christopher A. Seenan

**Affiliations:** 1 Centre for Living, School of Health and Life Sciences, Glasgow Caledonian University, Glasgow, UK; 2 Department of Medical Rehabilitation, Faculty of Health Science and Technology, College of Medicine, University of Nigeria, Enugu, Nigeria; 3 Department of Physiotherapy and Paramedicine, School of Health and Life Sciences, Glasgow Caledonain University, Glasgow, UK; 4 Vascular Surgery NHS Greater Glasgow and Clyde Health Board, Glasgow, UK; 5 Institute of Cardiovascular and Medical Sciences, University of Glasgow, Glasgow, UK; 6 Department of Sport, Exercise and Rehabilitation, Northumbria University, Newcastle, UK; 7 Institute of Applied Health Research, Glasgow Caledonian University, Glasgow, UK

**Keywords:** Peripheral arterial disease, Transcutaneous electrical nerve stimulation, Exercise, Physical activity, Patient-centred care, Behavioural change therapy, Intermittent claudication

## Abstract

**Background:**

Physical activity (PA) improves functional capacity and quality of life and provides secondary prevention benefits in individuals with peripheral arterial disease (PAD) and intermittent claudication (IC). However, pain and patient lack of knowledge are key barriers to the uptake of, and adherence to, PA recommendations. This trial will test the efficacy and feasibility of a non-invasive pain management intervention with and without patient education to improve PA in individuals with PAD and IC.

**Methods:**

This is a randomised, controlled assessor-blinded feasibility trial with four parallel groups. Eighty adults with PAD and IC will be randomly assigned 1:1:1:1 to Active TENS (transcutaneous electrical nerve stimulation), Placebo TENS, Active TENS + Patient education or Placebo TENS + Patient education groups. All groups will continue to receive usual care over the intervention period. Participants randomised to Active TENS will receive a TENS device (preset at 120 Hz, 200 μs) and will be instructed to use the device daily at home or elsewhere for 6 weeks with a patient-determined intensity of “strong but comfortable”. Placebo TENS group participants will receive the same model of TENS device and instructions for use as those in the active group, except that the stimulation dose will be safely altered to produce non-therapeutic, ineffective stimulation. Participants randomised to patient education will receive a one-off 3-h workshop of structured group education (four to five persons in each group) and three sets of twice-weekly phone calls. Efficacy outcomes will be assessed at baseline, after 6 weeks of intervention and at 3 months follow-up. Absolute claudication distance using the Gardner treadmill protocol will be assessed as the primary outcome. Secondary outcomes will assess initial claudication distance, daily PA and patient-reported outcomes including quality of life, pain self-efficacy, depression, disease perception and walking impairment pain intensity and quality. Feasibility outcomes will assess rates of recruitment, retention and adherence. Focus groups with participants at the end of the trial will explore the acceptability of the interventions.

**Discussion:**

This trial will determine the efficacy and feasibility of using a low-cost, CE-marked non-invasive pain management modality delivered with or without a patient-centred education intervention to improve PA in individuals with PAD and IC.

**Trial registration:**

ClinicalTrials.gov, NCT03204825. Registered on 2 July 2017.

**Electronic supplementary material:**

The online version of this article (10.1186/s13063-019-3307-6) contains supplementary material, which is available to authorized users.

## Background

Peripheral arterial disease (PAD) affects 2.7 million people in the UK [1]. The most common symptom that patients experience is intermittent claudication (IC), which is pain in the buttock, calf or thigh precipitated by exercise and relieved by rest [1]. The underlying cause of PAD is atherosclerosis, which leads to arterial stenosis and inadequate blood flow and tissue oxygen delivery during exercise [2–5]. Given the diffuse nature of atherosclerosis and the involvement of other arterial beds, patients with PAD and IC have a three to four times increased mortality compared to age and sex matched controls [6]. In addition IC has a major negative impact on patients’ mobility and quality of life [7, 8].

Patients with symptomatic PAD should receive the same secondary prevention management as patients with symptomatic coronary artery disease [9]. Improving daily physical activity (PA) is particularly important in individuals with IC, as lower PA levels have been recognised as a strong predictor of increased morbidity and mortality in this population [10]. Current National Institute for Health and Care Excellence (NICE) guidelines recommend the use of supervised exercise programmes, encouraging patients “to exercise to the point of maximal pain”, as first line treatment [11]. However, whilst supervised exercise programmes lead to a significant improvement in the absolute walking distances of patients with IC on a treadmill, it is unclear if this is sustained or leads to improvement in daily PA [12]. Furthermore, due to the resources required to deliver the recommended 3-month exercise programme (30–45 min three times weekly), supervised exercise programmes are not always routinely available to UK National Health Service (NHS) patients. In addition, time and travel challenges (compounded by background mobility issues) including costs tend to lead to low patient uptake and high attrition rates [13]. Therefore, investigating the feasibility of using low-cost, patient-centred interventions that can support increased PA is warranted.

Lack of self-efficacy, attributed to poor understanding of the disease and uncertainty regarding the importance of exercise, has been shown to be a major barrier to exercise uptake in this population [14]. Also, for patients with IC to maximise the benefits of improved walking ability and secondary prevention, they are recommended to exercise beyond the point when pain occurs, which represents another barrier to engagement in PA [15]. These barriers to pain and lack of knowledge underscore the importance of including pain management and patient education components in a low-cost, patient-centred intervention as key to enhancing uptake and adherence to exercise recommendations in individuals with PAD/IC [15, 16].

Our group recently developed and piloted Structured EDucation for Rehabilitation in Intermittent Claudication (SEDRIC) [17], a patient-centred education intervention with the specific aim of educating patients with IC about their condition, improving patient ownership and promoting self-managed walking. We found that in patients with IC (*n* = 14), treadmill walking distances (30%) and quality of life (32%) improved from baseline after 6 weeks of structured education. In addition, there was a trend for patients to increase their daily PA (approximately 8% changes from baseline).

Similarly, we have demonstrated in an experimental lower limb ischaemic pain model in healthy volunteers (*n* = 28) that transcutaneous electrical nerve stimulation (TENS), a low-cost, CE-marked non-invasive pain management device, significantly increased pain threshold, tolerance and endurance compared to placebo TENS [18]. Our extension proof-of-concept pilot study demonstrated that TENS, when applied to patients with IC exercising on a treadmill (*n* = 40), significantly improved absolute claudication distance (ACD) by 40% above placebo levels [19]. We have not assessed the ability of TENS to improve ACD when used during daily life.

Although patient-centred education (SEDRIC) and TENS have both demonstrated potential to improve PA in people with IC, the use of these components in combination has not previously been evaluated. Therefore, we do not know how potentially effective the combined intervention will be compared to each of the individual components. In addition, as part of the scaling process for complex intervention development, it is important to understand how the combined intervention can be feasibly delivered amongst patients with PAD and IC within the UK NHS. Equally key to informing the next stage of the project is the acceptability of the intervention as a whole or its individual components to patients with PAD and IC. Understanding these areas of uncertainty will address an important literature gap related to integrating two key components of pain management and patient education modalities in a patient-centred intervention to increase PA in individuals with PAD and IC.

To address these areas of uncertainty, we are undertaking a 2 × 2 factorial, assessor-blinded, parallel-group, placebo-controlled randomised trial of a *P*ain management and *P*atient *E*ducation for *P*hysical *A*ctivity in *I*ntermittent clau*D*ication (PrEPAID) intervention. The aim of the trial is to determine the efficacy and feasibility of a TENS device used with or without a patient-centred education programme to improve walking distances in patients with PAD and IC. The following research questions will be addressed:

- What is the efficacy of the TENS device used with or without a patient-centred education programme to improve walking distances in patients with PAD and IC?
- What is the feasibility (i.e. recruitment and retention rates, adherence, safety, sample size for a definitive trial) of conducting a definitive randomised controlled trial (RCT) of TENS with and without patient-centred education in patients with PAD and IC?
- How acceptable are TENS and patient-centred education as interventions on their own or in combination to patients with IC?

## Methods/design

### Trial design

**Fig. 1:**
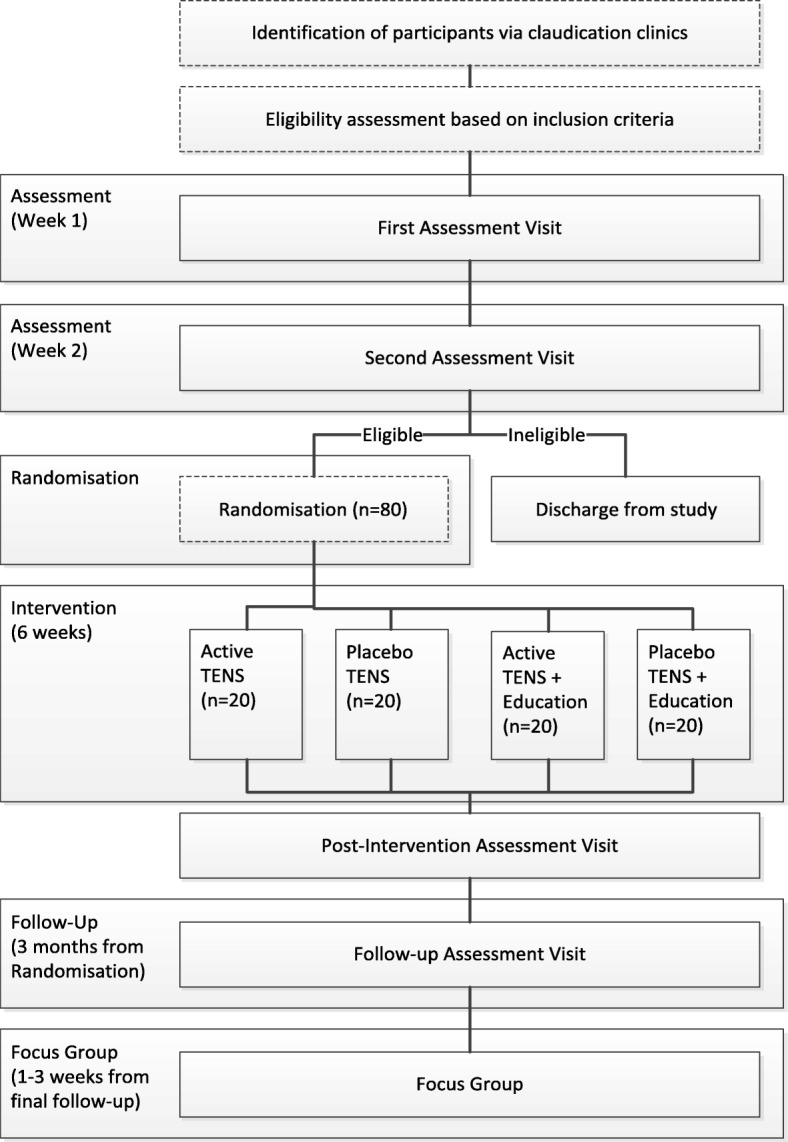
Flowchart for participant identification, inclusion, study design, interventions, assessments and follow-up

The PrEPAID trial is a 2 × 2 parallel group (TENS versus placebo TENS × patient education versus no additional education) feasibility RCT to compare the use of TENS against placebo TENS with and without a patient-centred education programme. Permuted block randomisation will be implemented to allocate patients to trial arms. The study design including patient inclusion, measurement and follow-up is shown in Fig. 1. Participants will be allocated to one of four groups (*n* = 20 per group): Active TENS, Active TENS + Patient education, Placebo TENS, Placebo TENS + Patient education. Active TENS/placebo and patient education interventions will be 6 weeks in duration, and all participants will receive secondary prevention therapy as recommended by NICE [11] including advice to exercise (usual care). Outcomes for all participants will be assessed at two points (at baseline and after 6 weeks of education programme and/or TENS (active or placebo). In addition, a subgroup of participants (those recruited in the first 8 months of recruitment) will be followed up 3 months post the 6-week intervention. All participants will be invited to attend a qualitative focus group to be conducted after final follow-up.

**Fig. 2:**
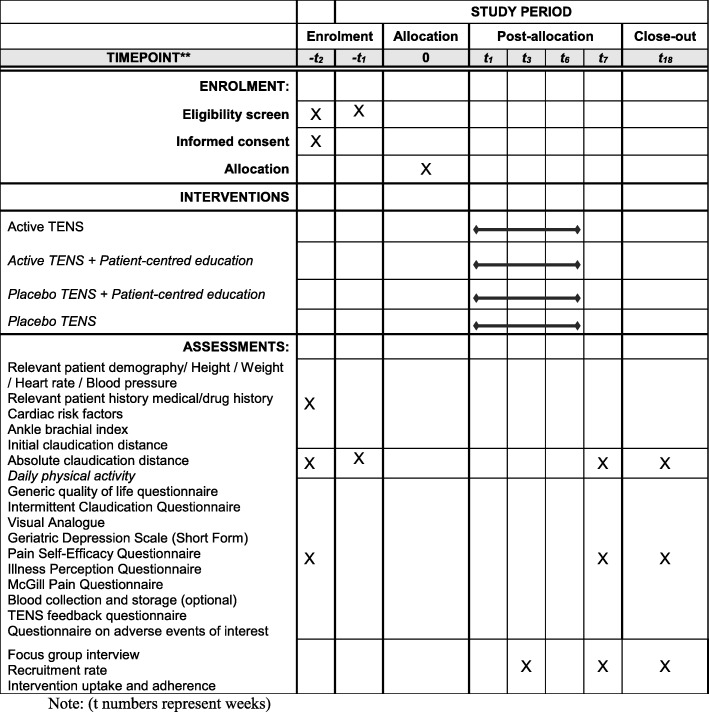
Standard Protocol Items: Recommendations for Interventional Trials (SPIRIT) figure (numbers beside *t* represent weeks)

The study protocol has been developed based on the Standard Protocol Items: Recommendations for Interventional Trials (SPIRIT). The SPIRIT figure (Fig. 2) summarises the planned study conduct, review, reporting and interpretation. The completed SPIRIT checklist is presented in Additional file 1. The Consolidated Standards of Reporting Trials (CONSORT) extension to pilot and feasibility trials [20] and the Template for Interventional Description and Replication (TIDieR) guidelines [21] will be followed in reporting the final outcome of this trial.

### Trial settings

This study is hosted at the Clinical Research Facility (CRF) of the Queen Elizabeth University Teaching Hospital, Glasgow, UK. Patients attending the NHS Greater Glasgow and Clyde vascular out-patient clinics will be invited to take part.

### Participant and eligibility criteria

Patients (aged 40–85 years) with a history of stable IC and an ankle-brachial pressure index (ABPI) ≤ 0.9 will be recruited. Participants would be included if they (1) have a clinical diagnosis of symptomatic PAD including resting ABPI ≤ 0.9 in at least one leg; (2) have had stable IC for ≥ 3 months; (3) have walking limited primarily by claudication; (4) are able to exercise on a treadmill; (5) are able to read and speak English to a level allowing satisfactory completion of the study procedures; (6) are able to provide written informed consent for participation. The following exclusion criteria will be applied to patients: (1) planned surgical or endovascular intervention for PAD within the next 3 months; (2) critical limb ischaemia; (3) the presence of any absolute contraindications to exercise testing/training as defined by the American College of Sports Medicine [22]; (4) previous experience of using TENS/ structured patient education for PAD; (5) contraindications to TENS (including epilepsy, dermatological conditions, indwelling electrical pumps/pacemakers); (6) inability to apply TENS independently (i.e. if a participant fails to demonstrate ability to apply TENS after receiving training); (7) patients who require walking aids including artificial limbs; (8) major surgery, myocardial infarction or stroke/transient ischaemic attack in the previous 6 months; (9) co-morbidities that cause pain or limit walking to a greater extent than IC (e.g. severe arthritis); (10) > 20% variation in baseline ACD on treadmill, taken at 2 weeks apart; (11) severe peripheral neuropathies above the ankle.

### Identification of participants and consent

Potential participants who are attending the vascular out-patient clinics within NHS Greater Glasgow and Clyde will be identified. They will be provided with a participant information sheet (Additional file 2), and contact details will be recorded on a study log. The nurse or other members of the study team will contact the patient, address any questions and arrange to meet. At this meeting, assuming the participant fulfils the trial eligibility criteria, informed consent (Additional file 3) may be taken by the nurse, or if the patient wishes, he/she will be given more time to consider participating in the trial. Potential participants who have recently attended the claudication clinic will also be contacted by post and sent a brief outline of the study and the participant information sheet. They will be asked to return a prepaid response slip stating whether they wish to be contacted further regarding the study. If problems arise with recruitment, then the option of using the Safe Haven or primary care records to help identify patients diagnosed with PAD and IC will be explored and appropriate approval obtained.

### Randomisation

Eligible and consented patients who have completed the baseline assessment and had ≤ 20% variation in ACD between the first and second visits will be randomly allocated to the trial arms. A central and independent randomisation facility (Internet-based randomisation system, the Interactive Web Response (IWR) system) will allocate the randomised therapy per patient. The IWR system, which will be based at the Robertson Institute University of Glasgow, will be available by web. A simple fixed block design (FBD) will be used to allocate patients to the groups. Randomisation outcome will be sent by email only to researchers involved in administering TENS and/or patient education. The outcome assessors and data analyst will be blinded after assignment to interventions.

### Sample size

For the primary outcome measure, at 80% power and a two-tailed 5% significance level, 16 participants per group will allow detection of an effect size of 1.0 standard deviation of ACD in the Active TENS group compared to the Placebo TENS control. Attrition rates in our previous pilot studies ranged from 7.1 [17] to 10% [19]. We will recruit 20 participants in each group, allowing for 20% attrition, and therefore aim to recruit 80 participants. If this effect size were applied to our separate pilot studies, this would provide the ability to detect a change of 169 m (TENS) [19] or 322 m (SEDRIC) [17] in our primary outcome measure of ACD. Indeed, in these studies, a sample size of 20 per group (TENS) and 14 per intervention group (SEDRIC) was sufficient to detect a significant difference in this outcome measure.

### Intervention procedures

#### Active TENS

Participants will be given a TENS device at the intervention visit. They will be instructed to use the device daily at home or elsewhere. They will be specifically advised to use the device prior to or during a challenging walk each day. Challenging walks could be for activities of daily living or planned exercise. Those with bilateral claudication will be advised to wear the device on the worst limb and to alternate it as symptoms fluctuate. The Active TENS group will receive high-frequency TENS calibrated to 120 Hz, 200 μs, and will be free to set the intensity to a “strong but tolerable” level [19]. An MTR+ Dolito TENS machine (EME Services Ltd., Manchester, UK) will be used.

##### Treatment schedule

Participants will be asked to wear the active TENS device every day as often as they can when they are awake and switch it on when they are standing/walking or about to engage in activity which they anticipate would trigger their IC pain. They will be instructed to switch it off after a maximum period of 1 h, for a rest period of at least 10–20 min, and to repeat this as often as warranted during daily activity.

##### Rationale for chosen TENS frequency

In a proof-of-concept study, high-frequency TENS (120 Hz) was found to increase the distance IC patients walked before reaching pain tolerance, and that high-frequency TENS (compared to low-frequency TENS) was more effective at prolonging the time to reach pain threshold [19].

##### Attachment

Patients will fit a TENS unit during wake periods and daily activity using two self-adhesive carbon rubber electrodes measuring 5 × 5 cm (StiMus® Hydrogel Premium Self-Adhesive Electrodes, EME Services Ltd.) attached to the TENS unit via the manufacturer’s leads. The area of pain reported by the participant would determine the electrode placement sites with the two electrodes to be placed at least 2 cm apart. Before it is provided to the patients for daily use, the TENS machine will be calibrated with a digital oscilloscope and tested manually by the research team.

#### Placebo TENS

Participants will receive the same model of TENS device and instructions for use as those in the active group except that the stimulation dose will be safely altered to produce non-therapeutic, ineffective stimulation (6 mA). This intensity setting will be locked off before the device is provided to the participants, and they will not be able to change it. This will allow the unit to be switched on with the appearance of a working unit. For the purposes of blinding, participants will be told that different dosages of TENS are being tested and for some of these dosages they might not feel anything even though the device is working. Indeed the placebo effect has been reported whilst using TENS in other conditions [23]; therefore, testing active TENS against placebo is recommended. All TENS units will be calibrated, checked and confirmed prior to being issued to participants. This method of achieving placebo has been successfully used in previous TENS trials [24–26].

#### Patient-centred education

The intervention for the groups receiving patient-centred education will be adopted from the successfully piloted SEDRIC study [17]: a one-off 3-h workshop of structured group education (four to five persons in each group) and three sets of twice-weekly phone calls. Two educators will implement the session. Training for educators will involve completion of the Diabetes Education and Self-Management for Ongoing and Newly Diagnosed (DESMOND) [27] core training; reading and demonstrating understanding of the SEDRIC [17] curriculum; and completion of at least two practice workshops that are quality assessed prior to delivering any sessions to patients. The aim of the structured education is to modify patients’ illness beliefs and perceptions about IC by educating them on disease pathology and management philosophy. After the workshop, each patient will be supported to set goals for walking based around a pedometer (Yamax SW-200 Digi-Walker pedometers) and daily steps, and to develop an action plan regarding how these goals will be met. Participants will be encouraged to repeat this process for each new walking goal through twice-weekly phone calls from the educators during which the progress, barriers and challenges are further discussed, and new walking goals will be set.

### Trial schedule

#### Informed consent

Written informed consent shall be obtained from each trial participant. The Research Nurse will explain the exact nature of the study in writing (by provision of the participant information sheet) and verbally, and will be responsible for consenting the participants. Trial participants will be informed that they are free to withdraw their consent from the study or study treatment at any time. Participants will be asked to attend the CRF at Queen Elizabeth University Hospital for a maximum of six occasions.

#### Visit 1: first assessment visit

At the first visit, participants will be assessed for eligibility, and those eligible will be requested to consent for further screening. Baseline outcome measurements will be conducted including weight/height/heart rate/blood pressure, ABPI, treadmill assessments, questionnaires and a blood sample. Participants will be fitted with the activPAL™ monitor and advised to wear it continuously for 7 days. They will be given instructions on how to use the activPAL and provided with a sleep diary to complete during the duration of wearing the activPAL.

#### Visit 2: second assessment visit (usually within 2 weeks after visit 1)

Participants will then attend for a second visit to undergo a second treadmill test and to return the activPAL and sleep diary. Only participants with ≤ 20% variation in ACD will continue in the trial. After this visit, eligible participants will be randomised and given a date on which to return for intervention (TENS/Placebo ± Patient education) as applicable. Recruitment and randomisation will be conducted in waves to allow groups to be formed for the education session.

#### Visit 3: intervention visit (within 3 weeks after randomisation)

Participants attend the clinic to receive the TENS device and training instructions for its daily use as required, plus or minus the patient education per randomisation group. Participants allocated to receive education will undergo a structured group education session (four to six persons per group) according to the SEDRIC procedure [17]. Before the education session, participants allocated to receive TENS will be provided with the device and instructions for its use. The use of the device will also be demonstrated, and patients will be shown how to put it on and remove it. Participants will try out the device to be sure that they understand the procedure.

#### Visit 4: post-intervention assessment visit (end of the 6-week intervention)

The outcome assessments and procedure followed in visit 1 will be repeated including questionnaires, treadmill protocol, blood sample collection and fitting of activPAL. The treadmill test will be conducted by investigators who are blinded to the participants’ group allocation. Participants will be given a prepaid envelope in which to return the activPAL monitor.

#### Visit 5: follow-up assessment visit (3 months post-randomisation)

All participants recruited within the first 8 months of recruitment will be invited to return for a 3 months follow-up visit. The outcome assessments and procedure followed in visits 1 and 4 will be repeated including questionnaires, treadmill protocol, blood sample collection and fitting of activPAL. The treadmill test will be conducted by investigators who are blinded to the participants’ group allocation. Participants will be given a prepaid envelope in which to return the activPAL monitor. We shall allow assessment visit windows of ±2 weeks.

#### Visit 6: focus group visit (usually 1–3 weeks after the final follow-up)

All participants will be invited to participate in a focus group discussion. The discussion sessions, lasting 1 h, will explore the acceptability of and satisfaction with the PrEPAID programme, components that were useful or not (in terms of helping them with PA) and participants’ suggestions for changes. Each focus group will consist of four to six participants and will be facilitated by an independent investigator. The number of focus groups to be conducted will be determined by data saturation, and the sessions will be audio-recorded and transcribed verbatim.

#### Laboratory tests

At visits 1, 4 and 5, 20 ml of blood will be taken from rested subjects. The samples will be spun and stored as per the standard operating procedure at the CRF at Queen Elizabeth University Hospital for future analysis of markers of angiogenesis and inflammatory response.

### Participant retention and withdrawal

All reasonable efforts, within the CRF local standard operating procedure, will be made to ensure optimum participant engagement and to reduce study attrition. However, the study involves an intention-to-treat analysis; therefore, if a patient does not apply the TENS or attend the education class (if randomised to this arm), he/she will continue to be followed up. Nonetheless, all participants will have the right to withdraw from the study at any stage. If the participant is willing to provide them, the reasons for withdrawal will be documented and any data already collected from that participant will be analysed.

### Outcome definitions

#### Efficacy outcomes

Measurements will be obtained at baseline, following the 6-week intervention and at 3 months follow-up. The primary efficacy outcome will be the treadmill-assessed absolute claudication distance (ACD) (metres) using the Gardner treadmill protocol [28].

Secondary efficacy outcomes will include initial claudication distance (metres) assessed by a treadmill exercise using the Gardner treadmill protocol [28]. Daily PA will be assessed via activPAL data outcomes: total number of (1) steps; (2) upright events; (3) walking events; (4) event-based claudication index (ratio of walking events to upright events) participants undertake in a day [29]. Three days of activPAL data at each time point shall be specified as minimum for including a patient’s activPAL data in the efficacy analysis.

Other secondary outcomes will assess patient-reported outcome measures. Disease-specific quality of life will be as assessed using the Intermittent Claudication Questionnaire [30]. Generic quality of life via the General Quality of Life Questionnaire (Short Form) [31], specifically the total item score as well as the two main scores (physical compound score and mental compound score), will be analysed. Pain intensity will assessed with the visual analogue scale, whilst pain quality will be recorded using the McGill Pain Questionnaire [32] 5 min after every treadmill test. Average pain intensity in the past 7 days will be recorded using the visual analogue scale [33]. Illness beliefs and psychosocial determinants of health and behaviour will be recorded using the Brief Illness Perception Questionnaire [34], the Geriatric Depression Scale: Short Form [35] and the Pain Self-Efficacy Questionnaire [36].

#### Feasibility and acceptability outcomes

Regarding recruitment rates, reasons for non-eligibility and non-recruitment of eligible patients will be recorded via the study screening log. Participants’ retention throughout the trial and reasons for withdrawal will be documented. Adverse events in all groups will be monitored, recorded, managed and followed up. Intervention uptake (log of TENS use and attendance at education) and acceptability of these interventions will be measured via a questionnaire. TENS blinding fidelity will be assessed via a TENS feedback questionnaire. Outcome completion rate for all outcomes (number of days the activPAL is worn, treadmill test completion, patient-reported outcome measures at each outcome time point) will be assessed. Acceptability of the intervention will be assessed through focus group discussions at end of follow-up.

### Recording and reporting of adverse events

We do not anticipate that the use of the CE-marked TENS device will result in any serious adverse events. Participants will be given prior information regarding the reporting of adverse events and measures to take including instructions to contact the research team via a dedicated phone line. At each study visit, participants will be specifically queried regarding the following adverse events of special interest: any case of itching, skin breakdown, mild electrical burn, other skin allergies or mild autonomic responses. Reported related adverse events will be documented in an applicable adverse event form.

#### Timing of outcome assessments

PA and patient-reported outcome measures will be assessed at baseline, at the end of the 6-week intervention (up to 2 weeks window) and 3 months post-randomisation (up to 2 weeks window). The recruitment, retention, outcome completion, intervention uptake and attrition rates will be assessed at the end of the study. Blinding and patients’ acceptability of TENS and ease of use will be assessed at the point of patient exit from the study. Further assessment regarding patients’ qualitative experience of the intervention will be conducted after 3 months post-randomisation (up to 2 weeks window).

### Statistics and data analysis

The Robertson Centre for Biostatistics, part of the Glasgow Clinical Trials Unit, a fully registered UK Clinical Research Network (UKCRN) Clinical Trials Unit, will manage the trial data. Statistical analysis will be led by the study senior statistician (JG) at the Institute for Applied Health Research, Glasgow Caledonian University; the statistician is blinded to group allocation. Data analysis will be performed following a detailed prespecified statistical analysis plan, which will be published separately.

In summary, an intention-to treat analysis will be performed for the primary outcome on all randomised patients, except those who withdraw consent for the use of their data [37, 38]. Baseline variables will be summarised using descriptive statistics. Also, the feasibility, acceptability, adverse events data and protocol and intervention adherence data will be summarised by randomised group and overall using descriptive statistics. Outcomes related to experience and perception via focus groups will be analysed by framework analysis [39, 40].

Comparisons will be undertaken to investigate the feasibility of studying the proposed outcomes for a definitive trial and to calculate estimates for the likely effect sizes and 95% confidence intervals (CIs). To determine the feasibility of conducting a definitive trial, inferential analysis will be conducted at 95% CI, and the *p* value will be set at *p* < 0.05. The change in the primary outcome will be compared between and within groups using Mann-Whitney *U* or Wilcoxon signed-rank tests (or their parametric equivalents) as applicable for between- and within-group comparisons. The log-rank method for pooled samples or substrata will be implemented where appropriate and when possible. Baseline participants’ variability will be controlled for using the analysis of covariance. Also, other secondary efficacy analyses will examine differences in the changes in activPAL outcomes, initial claudication distance, patient-reported outcomes and effect scores calculated using Mann-Whitney *U* or Wilcoxon signed-rank tests (or their parametric equivalents) as applicable for between- and within-group comparisons. Log-rank methods for pooled samples will be conducted where indicated. For the statistical analysis the software to be used is either SAS 9.2 for Windows (the SAS Institute, Cary, NC, USA) or SPSS Version 22.

### Data handling

#### Case report forms

An electronic case report form (e-CRF) will be used to collect study data. The e-CRF will be developed by the study Data Centre at the Robertson Centre for Biostatistics, University of Glasgow, and access to the e-CRF will be restricted, with only authorised site-specific personnel able to make entries or amendments to the patients’ data. It is the responsibility of the research team to ensure completion and to review and approve all data captured in the e-CRF.

All data handling procedures will be detailed in a study-specific data management plan. Data will be validated at the point of entry into the e-CRF and at regular intervals during the study. Data discrepancies will be flagged to the study site, and any data changes will be recorded in order to maintain a complete audit trail (reason for the change, date the change was made, who made the change).

#### Record retention

To enable evaluations and/or audits from regulatory authorities, the investigators will keep records, including the identity of all participants (sufficient information to link records), all original signed informed consent forms, serious adverse event forms, source documents and detailed records of treatment disposition in accordance with the Good Clinical Practice guidelines, local regulations or as specified in the Clinical Study Agreement, whichever specifies a longer retention time. Data will be retained at the Data Centre for a minimum of 5 years.

### Trial management

This research falls under the auspices of the clinical governance structure of Glasgow Caledonian University and NHS Greater Glasgow and Clyde CRF. The project is sponsored by Glasgow Caledonian University, and the Glasgow Caledonian University Research and Development Office will have responsibility for oversight, including audit of adherence to protocol and research governance standard operating procedures.

#### Trial Management Group

The trial will be coordinated from Glasgow Caledonian University by the Trial Management Group. This will consist of the co-investigators, NHS Greater Glasgow and Clyde CRF Research Nurse, the Robertson Centre for Biostatistics and the Glasgow Clinical Trial Unit. The Trial Management Group will be responsible for the overall management and completion of the project to timescales. The role of the group is to monitor all aspects of the conduct and progress of the trial, ensure that the protocol is adhered to and take appropriate action to safeguard participants and the quality of the trial itself. The group will meet bimonthly mainly via telephone conferences.

#### Trial Steering Committee

The Trial Steering Committee will utilise the strengths of diverse experts, including NHS Greater Glasgow and Clyde service users. This will help ensure that the research is relevant and accessible to a diverse audience. The committee will have an independent chair. Specifically, the committee will advise on the suitability of the interventions for the population group and design and participate in dissemination activities. The group members consist of the Chief Investigators (CS and JB), co-investigator (UA), two patient representatives, an expert in patient education interventions and PA behaviour change and an NHS management representative. The steering group will meet four times spread throughout the study with the aim of providing advice from a broad perspective.

#### Protocol amendments

Any change in the study protocol will require an amendment. Any proposed protocol amendments will be initiated by the Chief Investigators following discussion with the Trial Steering Committee, and any required amendment forms will be submitted to the ethics committee, funder, sponsor and NHS Greater Glasgow and Clyde Research and Development for approval as appropriate to their role. The Chief Investigators and the Trial Steering Committee will liaise with the study sponsor to determine whether an amendment is non-substantial or substantial. All amended versions of the protocol will be signed by the Chief Investigator and sponsor representative.

#### Insurance and indemnity

NHS indemnity is provided under the Clinical Negligence and Other Risks Indemnity Scheme and the Glasgow Caledonian University indemnity insurance.

#### Study reports

An annual progress report will be submitted to the funder, the Chief Science Office (CSO), Scotland, UK, the first being submitted 6 months from the date that all trial-related approvals are in place. Annual reports will be submitted to the ethics committee and sponsor, with the first submitted one year after the date that all trial-related approvals are in place. Recruitment data will, on a monthly basis, be uploaded to the UKCRN Portfolio database (and agreed successor to the database) through the mechanisms provided for the purpose, as part of the CSO requirement. Also, updated information on the outputs from the project shall be uploaded through the e-VAL system, which is now accessed through the ResearchFish website at https://researchfish.com/user/login?destination=awards. A final project report and other information and actions as required by the CSO as part of the project completion will be available and completed to the satisfaction of the CSO by the end of the funding period. Copies of all publications originating from this trial shall be provided to the CSO. The Chief Investigators and Project Management Group will produce all reports. All statistical reports will be produced by the Study Statistician from the Institute for Applied Health Research, Glasgow Caledonian University.

### Participants’ data protection

The data obtained from participants will remain confidential and stored securely at the Robertson Centre for Biostatistics University of Glasgow. The data are held in accordance with the Data Protection Act, which means that we keep it safely and cannot reveal it to other people without appropriate permission. The data held on the database will not be identifiable. In addition, PA data and basic demographic data will be kept on a password protected database on a secure server at Glasgow Caledonian University. The data held on the database will not be identifiable. This information collected may be used for further analysis by staff and students in the School of Health and Life Sciences at Glasgow Caledonian University at a later date.

#### Trial dissemination

The outcomes of the trials will be widely disseminated in journals and at scientific conferences.

## Discussion

Individuals with peripheral arterial disease (PAD) and intermittent claudication (IC) are at high risk of cardiovascular events, hospitalisation and death [41–44]. Encouraging physical activity (PA) in individuals with IC is of both clinical and public health importance. Supervised exercise programmes are effective and are the recommended therapy to improve walking distances, cardiovascular fitness and quality of life benefits in individuals with IC. However, supervised exercise programmes are not readily available to most patients with PAD and IC, and, when available, the programmes record low patient engagement and high attrition.

In two recent systematic reviews, we identified patients’ lack of disease understanding, uncertainty about exercise and the claudication pain as important barriers that prevent patients with PAD and IC from engaging in walking exercise [45], contributing to worse disease experience [46]. The results of these reviews underscored the importance of a self-management perspective to develop an intervention that concomitantly manages pain and educates patients about their disease pathology and the rationale of walking as a way of encouraging patients to adopt and adhere to the walking exercise recommendation. We also identified important intervention components through a series of systematic reviews, and we have conducted a series of laboratory, proof-of-concept and pilot studies underpinning both components of PrEPAID. We subsequently developed PrEPAID following the Medical Research Council (MRC) framework for complex interventions [47].

Whilst patient-centred education and TENs for pain management have both individually demonstrated potential to increase PA in individuals with PAD and IC, the use of these components together has not been evaluated. In line with the MRC framework for complex intervention development and evaluation, this trial will demonstrate the feasibility and potential benefit of TENS used with or without patient-centred education to improve PA in individuals with PAD and IC. This is an important consideration, since the two components of the intervention primarily target different important barriers to walking in this population. Findings will also be important in refining and tailoring the next phase of the intervention as well as in estimating sample size for the full RCT.

### Trial status

The study is ongoing at the time of submitting this manuscript (December 2018). This trial was using protocol version 2.0 (12 September 2017) at the time of this submission. Recruitment started in the Queen Elizabeth University Hospital on 17 May 2018 and is expected to be completed on 17 July 2019. The trial management committee manages and disseminates the protocol amendments.

## Additional files

Additional file 1: Completed SPIRIT checklist for PrEPAID trial. (DOC 382 kb)

Additional file 2: Participant information sheet for PrEPAID trial. (DOCX 190 kb)

Additional file 3: Participant consent form for PrEPAID trial. (DOCX 217 kb)

## Abbreviations

ABPI: Ankle-brachial pressure index
ACD: Absolute claudication distance
CRF: Clinical Research Facility
CSO: Chief Science Office
DESMOND: Diabetes Education and Self-Management for Ongoing and Newly Diagnosed
IC: Intermittent claudication
NHS: National Health Service
NICE: National Institute for Health and Care Excellence
PA: Physical activity
PAD: Peripheral arterial disease
PrEPAID: Pain management and Patient Education for Physical Activity in Intermittent clauDication
RCT: Randomised controlled trial
SEDRIC: Structured EDucation for Rehabilitation in Intermittent Claudication
TENS: Transcutaneous electrical nerve stimulation
