# Supplementary material for: Efficacy and Feasibility of Pain management and Patient Education for Physical Activity in Intermittent claudication (PrEPAID): protocol for a randomised controlled trial
Source: Trials. 2019 Apr 16;20:222. doi: 10.1186/s13063-019-3307-6 (PMC6469131; doi:10.1186/s13063-019-3307-6)
Supplement: Supplementary file 1 — Completed SPIRIT checklist for PrEPAID trial. (DOC 382 kb) [file 13063_2019_3307_MOESM1_ESM.doc]

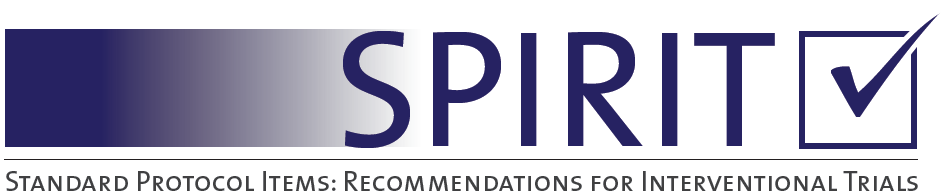


Additional file 1: PrEPAID Completed SPIRIT 2013 Checklist: Recommended items to address in a clinical trial protocol and related documents*

| Section/item | Item No | Description | Addressed on page number |
| --- | --- | --- | --- |
| **Administrative information** | | |  |
| Title | 1 | Efficacy and Feasibility of a Pain management and Patient Education for Physical Activity in Intermittent claudication (PrEPAID): protocol for a randomised controlled trial | ___1__________ |
| Trial registration | 2a | Trial register: ClinicalTrials.gov, ID: NCT03204825 | __3___________ |
|  | 2b | **Primary Registry and Trial Identifying Numbe**r: *ClinicalTrials.gov, ID: NCT03204825*  **Date of Registration in Primary Registry:** *Registered on 2 July 2017*.  Sponsor: Glasgow Caledonian University (Sponsor’s Protocol Number: 16-044).  **Source(s) of Monetary or Material Support:** *Chief Science Office, Scotland United Kingdom*  **Primary Sponsor:** *Glasgow Caledonian University*  **Secondary Sponsor(s):** NA  **Contact for Public Queries:** *Chris Seenan*  **Contact for Scientific Queries:** *Ukachukwu Abaraogu*  **Public Title: PrEPAID study**  **Scientific Title:** *Pain Management and Patient Education for Physical Activity in Intermittent*  *Claudication: Feasibility Randomised Controlled Trial*  **Countries of Recruitment:** *This study is hosted at the Clinical Research Facility of the Queen Elizabeth University Teaching Hospital, Glasgow, United Kingdom*  **Health Condition(s) or Problem(s) Studied:** Patients (aged 40-85 years) with a history of stable IC and an ABPI≤0.9 will be recruited.  **Intervention(s)**  **Key Inclusion and Exclusion Criteria**  Participants would be included if they: (1) have clinical diagnosis of symptomatic PAD including resting ABPI <0.9 in at least one leg; (2) have had stable IC for ≥3 months; (3) have walking limited primarily by claudication; (4) are able to exercise on a treadmill; (5) are able to read and speak English to a level allowing satisfactory completion of the study procedures; (6) are able to provide written informed consent for participation.  The following exclusion criteria will be applied to patients: (1) planned surgical or endovascular intervention for PAD within the next 3 months; (2) critical limb ischaemia; (3) the presence of any absolute contraindications to exercise testing/training as defined by the American College of Sports Medicine (ACSM); (4) previous experience of using TENS/ structured patient education for PAD; (5) contraindications to TENS (including epilepsy, dermatological conditions, indwelling electrical pumps/pacemakers); (6) inability to apply TENS independently (i.e. if a participant fails to demonstrate ability to apply TENS after receiving training); (7) patients who require walking aids including artificial limbs; (8) major surgery, myocardial infarction or stroke/ TIA in the previous 6 months; (9) co-morbidities that cause pain or limit walking to a greater extent than IC (e.g. severe arthritis); (10) >20% variation in baseline ACD on treadmill; taken at 2 weeks apart (11) severe peripheral neuropathies above the ankle.  **Study Type:** *The pain management and patient education for physical activity in individuals with intermittent claudication (PrEPAID) is a 2 x 2 parallel group (TENS versus placebo TENS x patient education versus no additional education) feasibility RCT*  **Date of First Enrolment:** *Enrolment started in the Queen Elizabeth University Hospital on May 17, 2018.*  **Target Sample Size:** *For the primary outcome measure, at 80% power and a two-tailed 5% significance level, 16 participants per group will allow detection of an effect size of 1.0 standard deviations of ACD in the active TENS group compared to placebo control. Attrition rates in our previous pilot studies ranged from 7.1%[16] to 10%[18]. We will recruit 20 participants in each group, allowing for 20% attrition, and therefore aim to recruit 80 participants. If this effect size were applied to our separate pilot studies, this would provide the ability to detect a change of 169m (TENS)[18] or 322m (SEDRIC)[16] in our primary outcome measure of ACD. Indeed, in these studies, a sample size of 20 per group (TENS) and 14 per intervention group (SEDRIC) was sufficient to detect a significant difference in this outcome measure.*    **Recruitment Status:**  The study is ongoing at the time of submitting this manuscript (September 2018).  **Primary Outcome(s)**  Primary efficacy outcomes will be treadmill assessed Absolute Claudication Distance (ACD)(m) using the Gardner treadmill protocol [28].  **Key Secondary Outcomes**  Secondary efficacy outcomes will include Initial Claudication Distance (ICD)(m) assessed by a treadmill exercise using the Gardner treadmill protocol[28]. Daily physical activity will be assessed via activPAL data outcomes: total number of i) steps; ii) upright events; iii) walking events; iv) event-based claudication index (ratio of walking events to upright events) participants undertake in a day [29]. Three days activPAL data at each time-point shall be specified as minimum for including a patient activPAL data in the efficacy analysis. | _____3  3________  3  24  3  NA  See reg. site  See reg. site  See reg site  8  8  See reg. site  See reg. Site  7  19  10  19  Page 15 paragraph 5  From page 15 last paragraph to page 161^st^ paragraph |
| Protocol version | 3 | **Date and version identifier:** *This trial was using protocol version 2.0 (September 2017) at the time of this submission.* | Page 23 paragraph 1 |
| Funding | 4 | **Sources and types of financial, material, and other support:** *The trial is funded by Chief Scientist Office (CSO) Scotland Translational grant award (TCS/16/55). The first author (AU) is being sponsored by the University of Nigeria through the TETFUND for a PhD being conducted at Glasgow Caledonian University. This protocol is part of a work towards the award of his PhD.* | Page 24 paragraph 2 |
| Roles and responsibilities | 5a | **Names, and affiliations of protocol contributors:** Ukachukwu Okoroafor Abaraogu Glasgow Caledonian University School of Health and Life Sciences Glasgow United Kingdom (Ukachukwu.Abaraogu@gcu.ac.uk; and University of Nigeria Department of Medical Rehabilitation, Enugu, Nigeria.Philippa Margaret Dall Glasgow Caledonian University School of Health and Life Sciences, Glasgow, United Kingdom. Julie Brittenden Vascular Surgery NHS Greater Glasgow and Clyde Health Board^6^Institute of Cardiovascular and Medical Sciences University of Glasgow. Wesley Stuart Vascular Surgery NHS Greater Glasgow and Clyde Health Board, Glasgow, United Kingdom. Garry Tew Northumbria University Department of Sport, Exercise and Rehabilitation, Newcastle, United Kingdom. Jon Godwin Institute of Applied Health Research Glasgow Caledonian University, Glasgow, United Kingdom  Christopher Andrew Seenan Glasgow Caledonian University School of Health and Life Sciences, Glasgow United Kingdom  **Roles of protocol contributors:** *AU drafted the manuscript. AUO, PMD, and CAS were involved in study conception and design. JB, GT, WS, and JG contributed to the design of the study. All authors contributed to manuscript revision, read and approved the final manuscript.* | Page 1  Pages 25 |
|  | 5b | **Name and contact information for the trial sponsor**: *Glasgow Caledonian University is the trial sponsor. Sponsor representative is Professor James Woodburn, Associate Dean Research/Director of Institute of Institute for Applied Health Glasgow Caledonian University. Email: Jim.Woodburn@gcu.ac.uk* | 23_____________ |
|  | 5c | **Role of study sponsor and funders, if any, in study design; collection, management, analysis, and interpretation of data; writing of the report; and the decision to submit the report for publication, including whether they will have ultimate authority over any of these activities:** *The funders have no role any role in the study design; collection, management, analysis, and interpretation of data; writing of this report; or decision to submit this report for publication.* | _23____________ |
|  | 5d | Composition, roles, and responsibilities of the coordinating centre, steering committee, endpoint adjudication committee, data management team, and other individuals or groups overseeing the trial, if applicable (see Item 21a for data monitoring committee):Trial management *This research will fall under the auspices of the clinical governance structure of Glasgow Caledonian University (GCU) and NHS GGC Clinical research facility. The project is sponsored by GCU and the GCU Research and Development Office will have responsibility for oversight, including audit of adherence to protocol and research governance Standard Operating Procedures.*  *Trial Management Group: The trial will be coordinated from Glasgow Caledonian University by the Trial Management Group. This will consist of the co-applicants, CRF research nurse, Robertson Centre for Biostatistics, and Glasgow Clinical Trial Unit. The trial Management Group will be responsible for the overall management and completion of the project to timescales. The role of the group is to monitor all aspects of the conduct and progress of the trial, ensure that the protocol is adhered to and take appropriate action to safeguard participants and the quality of the trial itself. The group will meet bimonthly mainly via telephone conferences.*  ***Trial steering committee (TSC):*** *The Steering committee will utilise the strengths of diverse experts, including NHS services users. This will help ensure that the research is relevant and accessible to a diverse audience. The committee will have an independent chair. Specifically, the committee will advise on the suitability of the interventions for the population group and design and participate in dissemination activities. The group members will consist of: the chief investigators; co-investigator (UA); 2 patient representatives; an expert in patient education interventions and PA behaviour change; and an NHS management representative. The steering group will meet four times spread throughout the study and aims to provide* advice from a broad perspective.  Protocol amendments: Any change in the study protocol will require an amendment. Any proposed protocol amendments will be initiated by the CIs following discussion with the TSC and any required amendment forms will be submitted to the ethics committee, funder, sponsor and NHS GGC R&D for approval as appropriate to their role. The CIs and the TSC will liaise with study sponsor to determine whether an amendment is non-substantial or substantial. All amended versions of the protocol will be signed by the CI and Sponsor representative. | Pages 19-20 |
| Introduction |  |  |  |
| Background and rationale | 6a | **Description of research question and justification for undertaking the trial, including summary of relevant studies (published and unpublished) examining benefits and harms for each intervention**: Peripheral Arterial disease (PAD) affects 2.7 million people in the UK[1]. The most common symptom that patients experience is Intermittent Claudication (IC), which is pain in the buttock, calf or thigh precipitated by exercise and relieved by rest[1]. The underlying cause of PAD is atherosclerosis, which leads to arterial stenosis, inadequate blood flow and tissue oxygen delivery during exercise[2][3][4][5]. Patients with IC have impaired quality of life[6][7]. Furthermore, due to the diffuse nature of atherosclerosis and the involvement of other arterial beds, they have 3-4 times increased mortality compared to age and sex matched controls[8].  Patients with symptomatic PAD should receive the same secondary prevention management as patients with symptomatic coronary artery disease. Improving daily physical activity (PA) is particularly important in individuals with IC as lower PA levels have been recognised as a strong predictor of increased morbidity and mortality in this population[9]. Current NICE guidelines recommend the use of supervised exercise programmes (SEPs), encouraging patients “to exercise to the point of maximal pain”, as first line treatment[10]. However, while SEPs lead to a significant improvement in the absolute walking distances of patients with IC on a treadmill, it is unclear if this is sustained or leads to improvement in daily PA[11]. Furthermore, due to the resources required to deliver the recommended 3 months exercise programme (30-45 minutes 3x weekly), SEPs are not always routinely available to NHS patients, and time and travel costs tend to lead to low patient uptake and high attrition rates[12]. Therefore, investigating the feasibility of using low-cost, patient-centred interventions that can support increased PA is warranted.  Lack of self-efficacy, attributed to poor understanding of the disease and uncertainty regarding the importance of exercise, has been shown to be a major barrier to exercise uptake in this population[13]. Similarly, for patients with IC to maximise benefits of improved walking ability and secondary prevention, exercising beyond the point when pain occurs is recommended, representing another barrier to engagement in PA[14]. These barriers of pain and lack of knowledge underscores the importance of including pain management and patient education components in a low low-cost, patient-centred intervention as key to enhancing uptake and adherence to exercise recommendations in individuals with PAD/IC[14][15].  Our group recently developed and piloted Structured EDucation for Rehabilitation in Intermittent Claudication (SEDRIC)[16], a patient-centred education intervention with the specific aim of educating patients with IC about their condition, improving patient ownership, and promoting self-managed walking. We found that in patients with IC (n=14), treadmill walking distances (30%) and quality of life (32%) improved from baseline after 6 weeks of structured education. In addition, there was a trend for patients to increase their daily PA (approx. 8% changes from baseline).  Similarly, we have demonstrated in an experimental lower limb ischaemic pain model in healthy volunteers (n=28) that Transcutaneous Electrical Nerve Stimulations (TENS), a low-cost, CE-marked non-invasive pain management device, significantly increased pain threshold, tolerance and endurance compared to placebo TENS[17]. Our extension proof-of-concept pilot study demonstrated that TENS when applied to patient with IC exercising on a treadmill (n=40) significantly improved absolute claudication distance (ACD) by 40% above placebo levels [18]. We have not assessed the ability of TENS to improve ACD when used during daily life.  Although patient-centred education (SEDRIC) and TENS have both demonstrated potential to improve PA in people with IC, the use of these components in combination has not previously been evaluated. Therefore, we do not know how potentially effective the combined intervention will be compared to each of the individual components. In addition, as part of the scaling process for complex intervention development, it is also important to understand how the combined intervention can be feasibility delivered among patients with PAD and IC within the UK NHS. Equally key to informing the next stage of the project is the acceptability of the intervention as a whole or its individual components to the patients with PAD and IC. Understanding these areas of uncertainty will address an important literature gap related to integrating two key components of pain management and patient education modalities in a patient-centred intervention to increase PA in individuals with IC.  To address the above areas of uncertainty, we are undertaking a 2 x 2 factorial, assessor-blinded, parallel-group, placebo-controlled randomized trial of a Pain management and Patient Education for Physical Activity in Intermittent clauDication (PrEPAID). The aim of the trial is to determine the efficacy and feasibility of transcutaneous electrical nerve stimulation device used within or without a patient centred education programme to improve walking distances in patients with PAD. The following research questions will be addressed.   - What is the efficacy of TENS device used within or without a patient centred education programme to improve walking distances in patients with PADand IC? - What is the feasibility (i.e. recruitment and retention rates, adherence, safety, sample size for a definitive trial, potential for effectiveness) of conducting a definitive RCT comparing TENS with and without patient-centred education patient with PAD and IC? - How acceptable are TENS and patient-centred education as interventions on their own or in combination to patients with IC? | Pages 4-7 |
|  | 6b | **Explanation for choice of comparators:** Although patient-centred education (SEDRIC) and TENS have both demonstrated potential to improve PA in people with IC, the use of these components in combination has not previously been evaluated. Therefore, we do not know how potentially effective the combined intervention will be compared to each of the individual components. In addition, as part of the scaling process for complex intervention development, it is also important to understand how the combined intervention can be feasibility delivered among patients with PAD and IC within the UK NHS. Equally key to informing the next stage of the project is the acceptability of the intervention as a whole or its individual components to the patients with PAD and IC. Understanding these areas of uncertainty will address an important literature gap related to integrating two key components of pain management and patient education modalities in a patient-centred intervention to increase PA in individuals with IC. | Pages 5-6 |
| Objectives | 7 | **Specific objectives or hypotheses:** *The aim of the trial is to determine the efficacy and feasibility of transcutaneous electrical nerve stimulation device used within or without a patient centred education programme to improve walking distances in patients with PAD* | 6-7__________ |
| Trial design | 8 | **Description of trial design including type of trial (eg, parallel group, crossover, factorial, single group), allocation ratio, and framework (eg, superiority, equivalence, noninferiority, exploratory):** *The pain management and patient education for physical activity in individuals with intermittent claudication (PrEPAID) is a 2 x 2 parallel group (TENS versus placebo TENS x patient education versus no additional education) feasibility RCT to compare use of TENS against placebo TENS with and without a patient-centred education programme.* | 7_____________ |
| Methods: Participants, interventions, and outcomes | | |  |
| Study setting | 9 | Description of study settings (eg, community clinic, academic hospital) and list of countries where data will be collected. Reference to where list of study sites can be obtained: *This study is hosted at the Clinical Research Facility of the Queen Elizabeth University Teaching Hospital, Glasgow, United Kingdom. Patients attending the NHS Greater Glasgow & Clyde vascular out-patient clinics will be invited to take part.* | 8_____________ |
| Eligibility criteria | 10 | **Inclusion and exclusion criteria for participants. If applicable, eligibility criteria for study centres and individuals who will perform the interventions (eg, surgeons, psychotherapists):** *Participants would be included if they: (1) have clinical diagnosis of symptomatic PAD including resting ABPI <0.9 in at least one leg; (2) have had stable IC for ≥3 months; (3) have walking limited primarily by claudication; (4) are able to exercise on a treadmill; (5) are able to read and speak English to a level allowing satisfactory completion of the study procedures; (6) are able to provide written informed consent for participation. The following exclusion criteria will be applied to patients: (1) planned surgical or endovascular intervention for PAD within the next 3 months; (2) critical limb ischaemia; (3) the presence of any absolute contraindications to exercise testing/training as defined by the American College of Sports Medicine (ACSM); (4) previous experience of using TENS/ structured patient education for PAD; (5) contraindications to TENS (including epilepsy, dermatological conditions, indwelling electrical pumps/pacemakers); (6) inability to apply TENS independently (i.e. if a participant fails to demonstrate ability to apply TENS after receiving training); (7) patients who require walking aids including artificial limbs; (8) major surgery, myocardial infarction or stroke/ TIA in the previous 6 months; (9) co-morbidities that cause pain or limit walking to a greater extent than IC (e.g. severe arthritis); (10) >20% variation in baseline ACD on treadmill; taken at 2 weeks apart (11) severe peripheral neuropathies above the ankle.* | _9____________ |
| Interventions | 11a | Interventions for each group with sufficient detail to allow replication, including how and when they will be administered: Intervention procedures **TENS:** Participants will be given TENS at the intervention visit. They will be instructed to use the device daily at home or elsewhere. They will be specifically advised to use the device prior to, or during, a challenging walk each day. Challenging walks could be for activities of daily living or planned exercise. For those with bilateral claudication they will be advised to wear the device on the worst limb and could alternate it as symptoms fluctuate. The active group will receive High Frequency-TENS calibrated to 120Hz, 200µs, and will be free to set intensity to a strong but tolerable’’ level[18]. A MTR+ Dolito TENS machine (EME Service Ltd., Manchester, United Kingdom) will be used.  Treatment schedule: Participants will be asked to wear active TENS everyday as often as they can when they are awake and switch it on when they are standing/ walking or about to engage in activity which they anticipate would trigger their IC pain. They will be instructed to switch it off after a maximum period of 1hr, for a rest period of at least 10-20 minutes, and to repeat this as often as warranted during daily activity.  Rationale for chosen TENS frequency: In a proof of concept study, HF-TENS was found to increase the distance IC patients walked before reaching pain tolerance, and that HF-TENS (compared to low frequency TENS) was more effective at prolonging the time to reach pain threshold.  Attachment: Patients will fit a TENS unit during wake periods and daily activity using 2 self-adhesive carbon rubber electrodes measuring 5 x 5 cm (StiMus® Hydrogel Premium Self-Adhesive Electrodes, EME Service Ltd., Manchester, United Kingdom) attached to the TENS unit via the manufacturers leads. The area of pain reported by the participant would determine the electrode placement sites with the 2 electrodes to be placed at least 2cm apart. Before it is handed out to the patients for daily use, the TENS machine will be calibrated with a digital oscilloscope and tested manually by the research team. **Placebo TENS:** Participants will receive the same model of TENS device and instructions for use as those in the active group except that the stimulation dose will be safely altered to produce non-therapeutic, ineffective stimulation (6mA). This intensity setting will be locked-off before the device will be handed to the participants and they will not be able to change it. This will allow the unit to be switched on with the appearance of a working unit. For the purposes of blinding, participants will be told that different dosages of TENS are being tested and for some of which they might not feel anything even though the device is working. Indeed the placebo effect has been reported while using TENS in other conditions[22], therefore, testing active TENS against placebo is advocated. All TENS units will be calibrated, checked and confirmed prior to being issued to participants. This method of achieving placebo has been successfully used in previous TENS trials [23][24][25]. **Patient-Centred Education:** The intervention for the groups receiving patient-centred education will be adopted from the successfully piloted SEDRIC study[26]: A one-off three-hour workshop of structured group education (4-5 persons in each group) and three 2-weekly phone calls. Two educators will implement the session. Training for educators will involve completion of the DESMOND[27] core training, reading and demonstrating understanding of the SEDRIC[26] curriculum, and completion of at least two practice workshops that are quality assessed prior to delivering any sessions to patients. The aim of the structured education is to modify patients’ illness beliefs and perceptions about IC by educating them on disease pathology and management philosophy. After the workshop, each patient will be supported to set goals for walking based around a pedometer (Yamax SW-200 Digi-Walker pedometers), and daily steps, and to develop an action plan regarding how these goals will be met. Participants will be encouraged to repeat this process for each new walking goal through 2-weekly phone calls from the educators during which the progress, barriers and challenges are further discussed, and new walking goals will be set. Trial schedule Informed consent: Written informed consent shall be obtained from each trial participant. The Research Nurse will explain the exact nature of the study in writing (by provision of the patient information sheet) and verbally, and will be responsible for consenting the participants. Trial participants will be informed that they are free to withdraw their consent from the study or study treatment at any time. Participants will be asked to attend the CRF a maximum of 6 occasions.  Visit 1- First Assessment Visit: At the first visit, participants will be assessed for eligibility and those eligible will be requested to consent for further screening. Baseline outcome measurements will be conducted including weight/height/heart rate/blood pressure, ABPI, treadmill assessments, questionnaires and taking a blood sample. Participants will be fitted with the activPAL^TM^ monitor and advised to wear this continuously for seven days. They will be given an instruction on how to use the activPAL, and provided with a sleep diary to complete during the duration of wearing the activPAL.  Visit 2- Second Assessment Visit (usually within 2 weeks after visit 1): Participants will then attend for a second visit to undergo a second Treadmill test, and to return the activPAL and sleep diary. Only participants with ≤20% variation in ACD will continue in the trial. After this visit eligible participants will be randomized, and given a date for them to return for intervention (TENS/Placebo ± Education) as applicable. Recruitment and randomisation will be conducted in waves to allow groups to be formed for the education session.  Visit 3- Intervention Visit (within 3 weeks after randomisation): Participants attend the clinic to receive the TENS and training instructions for its daily use as required, plus or minus the patient education per randomization group. Participants allocated to receive education will undergo a group structured education session (4-6 persons per group) according to the SEDRIC [26]. Before the education session, participants allocated to receive TENS will be provided with the device and instruction to use of the device. Also, the use of the device will be demonstrated, and patients shown how to put on and remove the device. Participants will try it out so on that to be sure they understand the procedure.  Visit 4- Post Intervention Assessment Visit (end of the 6-week intervention): Outcome assessments and procedure followed in Visit 1 will be repeated including questionnaires, treadmill protocol, blood sample collection and fitting of activPAL. The treadmill test will be conducted by investigators who are blinded to the participants’ group allocation. Participants will be given a prepaid envelope to return the activPAL monitor.  Visit 5- Follow-up Assessment Visit (3-months post-randomisation):All participants recruited within first eight months of recruitment will be invited to return for a 3-month follow-up visit. Outcome assessments and procedure followed in Visits 1 and 4 will be repeated including questionnaires, treadmill protocol, blood sample collection and fitting of activPAL. The treadmill test will be conducted by investigators who are blinded to the participants’ group allocation. Participants will be given a prepaid envelope to return the activPAL monitor. We shall allow assessment visit windows of ± 2 weeks.  Visit 6- Focus Group Visit (usually 1-3 weeks after the final follow-up): All participants will be invited to participate in a focus group discussion. The discussion sessions, lasting 1 hour, will explore the acceptability of and satisfaction with the PrEPAID programme, components that were useful or not (in terms of helping them with physical activity), and participants’ suggestions for changes. Each focus group, will consist of 4-6 participants, and will be facilitated by an independent investigator. The number of focus groups to be conducted will be determined by data saturation and the sessions will be audio-recorded and will be transcribed verbatim. | Page 10 paragraph 2 to page 14 |
|  | 11b | **Criteria for discontinuing or modifying allocated interventions for a given trial participant (eg, drug dose change in response to harms, participant request, or improving/worsening disease):** Participants’ retention and withdrawal: All reasonable efforts, within the CRF local SOP, will be made to ensure optimum participant engagement and to reduce study attrition. However, the study involves an intention to treat analysis and therefore if a patient does not apply the TENS or attend the education class (if randomised to this arm) they will continue to be followed up. Nonetheless, all participants will have the right to withdraw from the study at any stage. If willing, the reasons for withdrawal will be documented and any data already collected from that participant will be analysed. | _page 14 paragraph 2 |
|  | 11c | **Strategies to improve adherence to intervention protocols, and any procedures for monitoring adherence (eg, drug tablet return, laboratory tests)**  Participants’ retention and withdrawal: All reasonable efforts, within the CRF local SOP, will be made to ensure optimum participant engagement and to reduce study attrition. However, the study involves an intention to treat analysis and therefore if a patient does not apply the TENS or attend the education class (if randomised to this arm) they will continue to be followed up. Nonetheless, all participants will have the right to withdraw from the study at any stage. If willing, the reasons for withdrawal will be documented and any data already collected from that participant will be analysed. | Page 15 3^rd^ paragraph |
|  | 11d | Relevant concomitant care and interventions that are permitted or prohibited during the trial | _____________ |
| Outcomes | 12 | **Primary, secondary, and other outcomes, including the specific measurement variable (eg, systolic blood pressure), analysis metric (eg, change from baseline, final value, time to event), method of aggregation (eg, median, proportion), and time point for each outcome. Explanation of the clinical relevance of chosen efficacy and harm outcomes is strongly recommended** Outcome definitionsEfficacy outcomes: Measurements will be obtained at baseline, following six weeks intervention and at 3 months follow up.  Primary efficacy outcomes will be treadmill assessed Absolute Claudication Distance (ACD)(m) using the Gardner treadmill protocol [28].  Secondary efficacy outcomes will include Initial Claudication Distance (ICD)(m) assessed by a treadmill exercise using the Gardner treadmill protocol[28]. Daily physical activity will be assessed via activPAL data outcomes: total number of i) steps; ii) upright events; iii) walking events; iv) event-based claudication index (ratio of walking events to upright events) participants undertake in a day [29]. Three days activPAL data at each time-point shall be specified as minimum for including a patient activPAL data in the efficacy analysis. Feasibility and acceptability outcomes: Recruitment rates: reasons for non-eligibility and non-recruitment of eligible patients will be recorded via the study screening log. Participants’ retention throughout the trial and reasons for withdrawal will be documented. Adverse events in all groups will be monitored, recorded, managed and followed-up. Intervention uptake (log of TENS use and attendance at education) and acceptability of these interventions will be measured via a questionnaire. TENS blinding fidelity will be assessed via TENS feedback questionnaire. Outcome completion rate for all outcomes – number of days the activPAL is worn, treadmill test completion, and patient reported outcome measures (PROMS) at each outcome time point will be assessed. Acceptability of the intervention will be assessed through focus group discussions at end of follow up.  Other secondary outcomes will assess patient reported outcome measures (PROMs): Disease specific quality of life will be as assessed using the Intermittent Claudication Questionnaire (ICQ)[30]. Generic quality of life via the SF-36[31],specifically the total item score as well as the two main scores (physical compound score and mental compound score), will be analysed. Pain quality will be recorded using the McGill Pain Questionnaire (MPQ)[32] 5 minutes after every treadmill test. Average Pain intensity in the past 7 days will be recorded using a Visual Analogue Scale[33]. Illness beliefs and psychosocial determinants of health and behaviour will be recorded using the Brief Illness Perception Questionnaire (IPQ)[34], the Geriatric Depression Scale: Short Form (GDS-SF)[35], and the Pain Self-Efficacy Questionnaire (PSEQ)[36]. | Page 15 3^rd^ paragraph to page 16 2^nd^ & 3^rd^ paragraph |
| Participant timeline | 13 | Time schedule of enrolment, interventions (including any run-ins and washouts), assessments, and visits for participants. A schematic diagram is highly recommended (see Figure) | See SPIRIT diagram |
| Sample size | 14 | **Estimated number of participants needed to achieve study objectives and how it was determined, including clinical and statistical assumptions supporting any sample size calculations** Sample size For the primary outcome measure, at 80% power and a two-tailed 5% significance level, 16 participants per group will allow detection of an effect size of 1.0 standard deviations of ACD in the active TENS group compared to placebo control. Attrition rates in our previous pilot studies ranged from 7.1%[16] to 10%[18]. We will recruit 20 participants in each group, allowing for 20% attrition, and therefore aim to recruit 80 participants. If this effect size were applied to our separate pilot studies, this would provide the ability to detect a change of 169m (TENS)[18] or 322m (SEDRIC)[16] in our primary outcome measure of ACD. Indeed, in these studies, a sample size of 20 per group (TENS) and 14 per intervention group (SEDRIC) was sufficient to detect a significant difference in this outcome measure. | Page 10 1^ST^ paragraph |
| Recruitment | 15 | **Strategies for achieving adequate participant enrolment to reach target sample size**  Potential participants who are attending the vascular out-patients clinic within NHS GG&C will be identified. They will be provided with a participant information leaflet and contact details will be recorded on a study log. The nurse or other members of the study team will contact the patient, address any questions, and arrange to meet. At this meeting, assuming the participants fulfils the trial eligibility criteria informed consent may be taken by the nurse, or if the patient wishes, the patient will be given more time to consider participating in the trial. Potential participants who have recently attended the claudication clinic will also be contacted by post and sent a brief outline of the study and the patient information leaflet. They will be asked to return a pre-paid response slip stating whether they wish to be contacted further regarding the study. If problems arise with recruitment, then the option of using the Safe Haven or primary care records to help identify patients diagnosed with PAD and IC will be explored and appropriate approval obtained. | _Page 9 second paragraph |
| **Methods: Assignment of interventions (for controlled trials)** | | |  |
| Allocation: |  |  |  |
| Sequence generation | 16a | **Method of generating the allocation sequence (eg, computer-generated random numbers), and list of any factors for stratification. To reduce predictability of a random sequence, details of any planned restriction (eg, blocking) should be provided in a separate document that is unavailable to those who enrol participants or assign interventions:**  Eligible and consented patients who completed the baseline assessment and had ≤20% variation in ACD between the first and second visits will be randomly allocated to the trial arms. A central and independent randomisation facility (internet-based randomisation system, the Interactive Web Response (IWR) system) will allocate the randomised therapy per patient. The IWR system, based at the Data Centre, will be available by web. A simple fixed block design (FBD) will be used to allocated patients to the groups (PE+TENS, PE+Placebo TENS, TENS, Placebo TENS). Randomization outcome will be send by email only to researchers involved in administering TENS and/or patient education. Outcome assessors and data analyst will be blinded after assignment to interventions. | Page 9 last paragraph and page 10 first paragraph |
| Allocation concealment mechanism | 16b | **Mechanism of implementing the allocation sequence (eg, central telephone; sequentially numbered, opaque, sealed envelopes), describing any steps to conceal the sequence until interventions are assigned**  Eligible and consented patients who completed the baseline assessment and had ≤20% variation in ACD between the first and second visits will be randomly allocated to the trial arms. A central and independent randomisation facility (internet-based randomisation system, the Interactive Web Response (IWR) system) will allocate the randomised therapy per patient. The IWR system, based at the Data Centre, will be available by web. A simple fixed block design (FBD) will be used to allocated patients to the groups (PE+TENS, PE+Placebo TENS, TENS, Placebo TENS). Randomization outcome will be send by email only to researchers involved in administering TENS and/or patient education. Outcome assessors and data analyst will be blinded after assignment to interventions | Page 9 last paragraph and page 10 first paragraph |
| Implementation | 16c | **Who will generate the allocation sequence, who will enrol participants, and who will assign participants to interventions**  Eligible and consented patients who completed the baseline assessment and had ≤20% variation in ACD between the first and second visits will be randomly allocated to the trial arms. A central and independent randomisation facility (internet-based randomisation system, the Interactive Web Response (IWR) system) will allocate the randomised therapy per patient. The IWR system, based at the Data Centre, will be available by web. A simple fixed block design (FBD) will be used to allocated patients to the groups (PE+TENS, PE+Placebo TENS, TENS, Placebo TENS). Randomization outcome will be send by email only to researchers involved in administering TENS and/or patient education. Outcome assessors and data analyst will be blinded after assignment to interventions | Page 9 last paragraph and page 10 first paragraph |
| Blinding (masking) | 17a | **Who will be blinded after assignment to interventions (eg, trial participants, care providers, outcome assessors, data analysts), and how**  Randomization outcome will be send by email only to researchers involved in administering TENS and/or patient education. Outcome assessors and data analyst will be blinded after assignment to interventions. | page 10 first paragraph |
|  | 17b | If blinded, circumstances under which unblinding is permissible, and procedure for revealing a participant’s allocated intervention during the trial | NA |
| **Methods: Data collection, management, and analysis** | | |  |
| Data collection methods | 18a | **Plans for assessment and collection of outcome, baseline, and other trial data, including any related processes to promote data quality (eg, duplicate measurements, training of assessors) and a description of study instruments (eg, questionnaires, laboratory tests) along with their reliability and validity, if known. Reference to where data collection forms can be found, if not in the protocol** Efficacy outcomes: Measurements will be obtained at baseline, following six weeks intervention and at 3 months follow up.  Primary efficacy outcomes will be treadmill assessed Absolute Claudication Distance (ACD)(m) using the Gardner treadmill protocol [28].  Secondary efficacy outcomes will include Initial Claudication Distance (ICD)(m) assessed by a treadmill exercise using the Gardner treadmill protocol[28]. Daily physical activity will be assessed via activPAL data outcomes: total number of i) steps; ii) upright events; iii) walking events; iv) event-based claudication index (ratio of walking events to upright events) participants undertake in a day [29]. Three days activPAL data at each time-point shall be specified as minimum for including a patient activPAL data in the efficacy analysis. Feasibility and acceptability outcomes: Recruitment rates: reasons for non-eligibility and non-recruitment of eligible patients will be recorded via the study screening log. Participants’ retention throughout the trial and reasons for withdrawal will be documented. Adverse events in all groups will be monitored, recorded, managed and followed-up. Intervention uptake (log of TENS use and attendance at education) and acceptability of these interventions will be measured via a questionnaire. TENS blinding fidelity will be assessed via TENS feedback questionnaire. Outcome completion rate for all outcomes – number of days the activPAL is worn, treadmill test completion, and patient reported outcome measures (PROMS) at each outcome time point will be assessed. Acceptability of the intervention will be assessed through focus group discussions at end of follow up.  Other secondary outcomes will assess patient reported outcome measures (PROMs): Disease specific quality of life will be as assessed using the Intermittent Claudication Questionnaire (ICQ)[30]. Generic quality of life via the SF-36[31],specifically the total item score as well as the two main scores (physical compound score and mental compound score), will be analysed. Pain quality will be recorded using the McGill Pain Questionnaire (MPQ)[32] 5 minutes after every treadmill test. Average Pain intensity in the past 7 days will be recorded using a Visual Analogue Scale[33]. Illness beliefs and psychosocial determinants of health and behaviour will be recorded using the Brief Illness Perception Questionnaire (IPQ)[34], the Geriatric Depression Scale: Short Form (GDS-SF)[35], and the Pain Self-Efficacy Questionnaire (PSEQ)[36]. | From page 15 4^th^ paragraph to all of page 16 |
|  | 18b | **Plans to promote participant retention and complete follow-up, including list of any outcome data to be collected for participants who discontinue or deviate from intervention protocols**  Participants’ retention and withdrawal: All reasonable efforts, within the CRF local SOP, will be made to ensure optimum participant engagement and to reduce study attrition. However, the study involves an intention to treat analysis and therefore if a patient does not apply the TENS or attend the education class (if randomised to this arm) they will continue to be followed up. Nonetheless, all participants will have the right to withdraw from the study at any stage. If willing, the reasons for withdrawal will be documented and any data already collected from that participant will be analysed. | Page 15 3^rd^ paragraph |
| Data management | 19 | Plans for data entry, coding, security, and storage, including any related processes to promote data quality (eg, double data entry; range checks for data values). Reference to where details of data management procedures can be found, if not in the protocol Data handling Case report forms: An electronic case report form (e-CRF) will be used to collect study data. The e-CRF will be developed by the study Data Centre at the Robertson Centre for Biostatistics, University of Glasgow and access to the e-CRF will be restricted, with only authorised site-specific personnel able to make entries or amendments to the patients’ data. It is the responsibility of the research team to ensure completion and to review and approve all data captured in the e-CRF.  All data handling procedures will be detailed in a Study Specific Data Management Plan. Data will be validated at the point of entry into the e-CRF and at regular intervals during the study. Data discrepancies will be flagged to the study site and any data changes will be recorded in order to maintain a complete audit trail (reason for change, date change made, who made change).  Record retention: To enable evaluations and/or audits from regulatory authorities, the investigators will keep records, including the identity of all participants (sufficient information to link records), all original signed informed consent forms, serious adverse event forms, source documents, and detailed records of treatment disposition in accordance with ICH GCP, local regulations, or as specified in the Clinical Study Agreement, whichever is longer. Data will be retained at the Data Centre for a minimum of 5 years. | Page 19 paragraphs 1-3 |
| Statistical methods | 20a | Statistical methods for analysing primary and secondary outcomes. Reference to where other details of the statistical analysis plan can be found, if not in the protocol  The Robertson Centre for Biostatistics, part of the Glasgow Clinical Trials Unit, a fully registered UK CRN Clinical Trials Unit, will manage trial data. Statistical analysis will be led by the study senior statistician (JG), at the Institute for Applied Health Research, Glasgow Caledonian University who is blinded to group allocation. Data analysis will be performed following a detailed pre-specified statistical analysis plan, which will be published separately  In summary, an intention-to treat analysis will be performed for the primary outcome on all randomized patients, except those who withdraw consent for the use of their data[37][38]. Baseline variables will be summarized using descriptive statistics. Also the feasibility, acceptability, adverse events data and protocol and intervention adherence data will be summarised by randomised group and overall using descriptive statistics. Outcomes related to experience and perception via focus groups will be analysed by framework analysis[39][40].  Comparisons will be undertaken to investigate the feasibility of studying the proposed outcomes for definitive trial and to calculate estimates for the likely effect sizes and 95% confidence intervals. To determine the feasibility of conducting a definitive trial, inferential analysis will be conducted at 95% CI, and p-value will be set at p<0.05. The change in the primary outcome will be compared between and within groups using Mann-Whitney U or Wilcoxon signed-rank tests (or their parametric equivalents) as applicable for between- and within-group comparisons. The log-rank method for pooled samples or sub-strata will be implemented where appropriate and when possible. Baseline participants’ variability will be controlled for using the analysis of co-variance. Also, other secondary efficacy analysis will examine differences in the changes in activPAL outcomes, ICD, SF-36 and ICQ scores, MPQ score, IPQ, GDS-SF, PSEQ, and effect scores calculated using Mann-Whitney U or Wilcoxon signed-rank tests (or their parametric equivalents) as applicable for between and within-group comparisons. Log-rank methods for pooled samples will be conducted where indicated. Software for statistical analysis: The statistical software to be used is either SAS 9.2 for Windows, Cary, NC, USA or SPSS Version 22. | From Page 17 last paragraph to Page 18 |
|  | 20b | **Methods for any additional analyses (eg, subgroup and adjusted analyses)**  The log-rank method for pooled samples or sub-strata will be implemented where appropriate and when possible. Baseline participants’ variability will be controlled for using the analysis of co-variance. Also, other secondary efficacy analysis will examine differences in the changes in activPAL outcomes, ICD, SF-36 and ICQ scores, MPQ score, IPQ, GDS-SF, PSEQ, and effect scores calculated using Mann-Whitney U or Wilcoxon signed-rank tests (or their parametric equivalents) as applicable for between and within-group comparisons. Log-rank methods for pooled samples will be conducted where indicated. Software for statistical analysis. | Page 18 paragraph 2 lines 7 to 15 |
|  | 20c | **Definition of analysis population relating to protocol non-adherence (eg, as randomised analysis), and any statistical methods to handle missing data (eg, multiple imputation)**  …, the study involves an intention to treat analysis and therefore if a patient does not apply the TENS or attend the education class (if randomised to this arm) they will continue to be followed up. Nonetheless, all participants will have the right to withdraw from the study at any stage. If willing, the reasons for withdrawal will be documented and any data already collected from that participant will be analysed. | Page 15 3^rd^ paragraph lines 3-8 |
| **Methods: Monitoring** | | |  |
| Data monitoring | 21a | **Composition of data monitoring committee (DMC); summary of its role and reporting structure; statement of whether it is independent from the sponsor and competing interests; and reference to where further details about its charter can be found, if not in the protocol. Alternatively, an explanation of why a DMC is not needed** Trial management This research will fall under the auspices of the clinical governance structure of Glasgow Caledonian University (GCU) and NHS GGC Clinical research facility. The project is sponsored by GCU and the GCU Research and Development Office will have responsibility for oversight, including audit of adherence to protocol and research governance Standard Operating Procedures.  Trial Management Group: The trial will be coordinated from Glasgow Caledonian University by the Trial Management Group. This will consist of the co-applicants, CRF research nurse, Robertson Centre for Biostatistics, and Glasgow Clinical Trial Unit. The trial Management Group will be responsible for the overall management and completion of the project to timescales. The role of the group is to monitor all aspects of the conduct and progress of the trial, ensure that the protocol is adhered to and take appropriate action to safeguard participants and the quality of the trial itself. The group will meet bimonthly mainly via telephone conferences.  Trial steering committee (TSC): The Steering committee will utilise the strengths of diverse experts, including NHS services users. This will help ensure that the research is relevant and accessible to a diverse audience. The committee will have an independent chair. Specifically, the committee will advise on the suitability of the interventions for the population group and design and participate in dissemination activities. The group members consist of: the chief investigators (CS and JB); co-investigator (UA); 2 patient representatives; an expert in patient education interventions and PA behaviour change; and an NHS management representative. The steering group will meet four times spread throughout the study and aims to provide advice from a broad perspective | From page 19 last paragraphs to page 20 paragraphs 1-3. |
|  | 21b | Description of any interim analyses and stopping guidelines, including who will have access to these interim results and make the final decision to terminate the trial | NA |
| Harms | 22 | **Plans for collecting, assessing, reporting, and managing solicited and spontaneously reported adverse events and other unintended effects of trial interventions or trial conduct** Recording and reporting of Adverse EventsWe do not anticipate that the use of the CE-marked TENS device will result in any serious adverse events. Participants will be given prior information regarding the reporting of adverse events and measure to take including instructions to contact the research team via a dedicated phone line. Participants will be specifically queried regarding the following adverse events of special interest (any case of itching, skin breakdown, mild electrical burn, other skin allergies, or mild autonomic responses) at each study visit, and reported related adverse events will be documented in an applicable adverse event form. | Page 17 first paragraph |
| Auditing | 23 | **Frequency and procedures for auditing trial conduct, if any, and whether the process will be independent from investigators and the sponsor**  An annual progress report will be submitted to the funder, the Chief Scientist Office (CSO), Scotland United Kingdom, the first being submitted 6 months from the date that all trial related approvals are in place. Annual reports will be submitted to the ethics committee and sponsor with the first submitted one year after the date that all trial related approvals are in place. Recruitment data will, on a monthly basis, be uploaded to the UKCRN Portfolio database (and agreed successor to the database) through the mechanisms provided for the purpose, as part of CSO requirement. Also, updated information on the outputs from the project shall be uploaded through the e-VAL system, which is now accessed through the ResearchFish website - <https://www.researchfish.com/>. A final project report and other information and actions as required by CSO as part of the project completion will be available and completed to the satisfaction of CSO by the end of the funding period. Copies of all publications originating from this trial shall be provided to CSO. The Chief Investigators and Project Management Group will produce all reports. All statistical reports will be produced by the Study Statistician from the Institute for Applied Health Research, Glasgow Caledonian University. | Page 21 last paragraph |
| Ethics and dissemination | | |  |
| Research ethics approval | 24 | **Plans for seeking research ethics committee/institutional review board (REC/IRB) approval**  **Ethics approval and consent to participate**: The study protocol was approved by the West of Scotland Research Ethic Committee 4 (17/WS/0094), and the NHS Greater Glasgow and Clyde Clinical Research and Development (NHS GGC R&D) (GN16CE378). | Page 24 2^nd^ paragraph |
| Protocol amendments | 25 | **Plans for communicating important protocol modifications (eg, changes to eligibility criteria, outcomes, analyses) to relevant parties (eg, investigators, REC/IRBs, trial participants, trial registries, journals, regulators)**  Protocol amendments: Any change in the study protocol will require an amendment. Any proposed protocol amendments will be initiated by the CIs following discussion with the TSC and any required amendment forms will be submitted to the ethics committee, funder, sponsor and NHS GGC R&D for approval as appropriate to their role. The CIs and the TSC will liaise with study sponsor to determine whether an amendment is non-substantial or substantial. All amended versions of the protocol will be signed by the CI and Sponsor representative. | From page 20 last paragraph to page 21 first paragraph |
| Consent or assent | 26a | **Who will obtain informed consent or assent from potential trial participants or authorised surrogates, and how (see Item 32)**  Written informed consent shall be obtained from each trial participant. The Research Nurse will explain the exact nature of the study in writing, provision of patient information sheet, and verbally and will be responsible for consenting the participants. Trial participants will be informed that they are free to withdraw their consent from the study or study treatment at any time | Page 24 last paragraph |
|  | 26b | Additional consent provisions for collection and use of participant data and biological specimens in ancillary studies, if applicable | NA |
| Confidentiality | 27 | **How personal information about potential and enrolled participants will be collected, shared, and maintained in order to protect confidentiality before, during, and after the trial**  Participants’ data protection  The data obtained from participants will remain confidential and stored securely at the Robertson Centre for Biostatistics University of Glasgow. The data are held in accordance with the Data Protection Act, which means that we keep it safely and cannot reveal it to other people, without appropriate permission. The data held on the database will not be identifiable. In addition, physical activity data and basic demographic data will be kept on a password protected database on a secure server at Glasgow Caledonian University. The data held on the database will not be identifiable. This information collected may be used for further analysis by staff and students in the School of Health & Life Sciences at Glasgow Caledonian University at a later date. | Page 21 last paragraph to page 22 2nd paragraph |
| Declaration of interests | 28 | **Financial and other competing interests for principal investigators for the overall trial and each study site**  **Competing interest:** The authors declare that they have no competing interests | Page 24 last paragraph |
| Access to data | 29 | **Statement of who will have access to the final trial dataset, and disclosure of contractual agreements that limit such access for investigators**  **Availability of data and materials**: Anonymous copy of the final datasets underlying publications resulting from this trial will be shared upon reasonable and approved request. Request may be made through email to the PI, and can only be made upon meeting the terms and conditions for the ethics approval of this trial. | Page 24 last paragraph |
| Ancillary and post-trial care | 30 | Provisions, if any, for ancillary and post-trial care, and for compensation to those who suffer harm from trial participation | NA |
| Dissemination policy | 31a | **Plans for investigators and sponsor to communicate trial results to participants, healthcare professionals, the public, and other relevant groups (eg, via publication, reporting in results databases, or other data sharing arrangements), including any publication restrictions**  Trial dissemination: The outcomes of the trials will be widely disseminated in journals and scientific conference. | Page 22 paragraph 2 |
|  | 31b | **Authorship eligibility guidelines and any intended use of professional writers**  **Authors’ contributions:** AU drafted the manuscript. AU, PD, and CS were involved in study conception and design. JB, GT, WS, and JG contributed to the design of the study. All authors contributed to manuscript revision, read and approved the final manuscript. | Page 25 1^st^ paragraph |
|  | 31c | Plans, if any, for granting public access to the full protocol, participant-level dataset, and statistical code | NA |
| Appendices |  |  |  |
| Informed consent materials | 32 | Model consent form and other related documentation given to participants and authorised surrogates | Provided at part of supplementary files |
| Biological specimens | 33 | **Plans for collection, laboratory evaluation, and storage of biological specimens for genetic or molecular analysis in the current trial and for future use in ancillary studies, if applicable**  Laboratory tests: 20mls of blood will be taken from rested subjects at visits 1, 4 and 5, spun and stored as per the standard operating procedure (SOP) at the CRF at QEUH for future analysis of markers of angiogenesis and inflammatory response. | Page 15 2^nd^ paragraph |

*It is strongly recommended that this checklist be read in conjunction with the SPIRIT 2013 Explanation & Elaboration for important clarification on the items. Amendments to the protocol should be tracked and dated. The SPIRIT checklist is copyrighted by the SPIRIT Group under the Creative Commons “[Attribution-NonCommercial-NoDerivs 3.0 Unported](http://www.creativecommons.org/licenses/by-nc-nd/3.0/)” license.
