# Supplementary material for: Efficacy and Feasibility of Pain management and Patient Education for Physical Activity in Intermittent claudication (PrEPAID): protocol for a randomised controlled trial
Source: Trials. 2019 Apr 16;20:222. doi: 10.1186/s13063-019-3307-6 (PMC6469131; doi:10.1186/s13063-019-3307-6)
Supplement: Supplementary file 2 — Participant information sheet for PrEPAID trial. (DOCX 190 kb) [file 13063_2019_3307_MOESM2_ESM.docx]

**Additional File 2: PARTICIPANT INFORMATION SHEET**

**Project Title: Pain Management and Patient Education in people with Intermittent Claudication**

We would like to invite you to take part in a research study. Before you decide you need to understand why the research is being done and what it would involve for you. Please take time to read the following information carefully. Talk to others about the study if you wish. Ask us if there is anything that is not clear or if you would like more information.

**Who is conducting the research?**

The research is being performed at the Queen Elizabeth University Hospital. It involves researchers from Glasgow Caledonian University, University of Glasgow, Northumbria University, and NHS Greater Glasgow and Clyde.

**What is the purpose of the study?**

You have been invited to take part in this project as you have been diagnosed with Peripheral Arterial Disease (PAD). Patients with peripheral arterial disease have hardening and narrowing of their leg arteries. This leads to pain on walking due to insufficient blood reaching the leg muscles during exercise such as walking. The findings from a small study suggest that use of an electrical current applied to the muscle may reduce pain and allow patients to walk further on a treadmill. The electrical current is safely delivered through a machine called a Transcutaneous Electrical Nerve Stimulation (TENS)

In this study we would like to see if the TENS machine when used by you at home during normal daily activities and exercise can lead to an improvement in the distance you can walk. We also want to look into the possible benefit of an educational programme.

**Why have I been chosen?**

You have been diagnosed as having PAD and experience pain in your leg when walking.

**Do I have to take part?**

No. It is up to you to decide if you wish to take part in this study. You may take as long as you wish to decide. If you do decide to take part, we will require you to sign a consent form stating that you fully understand the study and have had the opportunity to ask questions. A decision to withdraw at any time, or a decision not to take part, will not affect the standard of care you receive or your future treatment or your rights/ relationships with Glasgow Caledonian University or NHS Greater Glasgow and Clyde.

**What will I have to do if I take part?**

If you agree to take part in the study, you will be allocated by chance to 1) a TENS machine which you can adjust the intensity or a TENS machine with intensity fixed, which you will use over a six week period and 2) you will be allocated by chance to an educational programme or normal advice.

You will be shown how to use the TENS machine.

You will be asked to attend the hospital on five occasions, with the option of one additional visit.

***During the first visit*** (lasting approximately 1 hour) you will be asked to walk on a treadmill under supervision. We will ask you to walk on until you reach the point where you feel that you cannot walk any further due to the pain in your leg(s). How long this will take will depend on the severity of your symptom but we anticipate no longer then 10-15minutes. You will also be requested to fill in a questionnaire which asks you about how PAD affects your quality of life. You will also be asked to provide an optional blood sample (2 table spoons optional).

Between the first and second visits you will be asked to wear a small, inconspicuous physical activity monitor on your thigh for seven days.

***During the second visit*** (lasting 25minutes) you will be asked to walk on the treadmill and complete one questionnaire. The treadmill procedure will be the same as in the first visit.

If there is a large difference in the distances you walked on the treadmill between visit 1 and 2 you will not be able to continue in the study. If there is not a large difference we will allocate you by chance (like tossing a coin) to receive either TENS with adjustable intensity or TENS with fixed intensity and to education session or normal advice

***At the third visit*** (1 hour or 4 hours maximum) you will receive the intervention. You will be given a TENS machine and shown how to put it on and use it. We are testing two different settings in the study, and you will be told which settings to use. You will then be asked to use the TENS device at home and in daily activities for 6 weeks. Half the people taking part in the study will be asked to attend the hospital for an education session. This will last an extra 3 hours

***The fourth visit*** (lasting approximately 1 hour) will be a repeat of the first visit where you will complete the questionnaires, treadmill test and blood sample (optional). You will be asked to wear a small, inconspicuous physical activity monitor on your thigh for the seven days following this visit.

***Depending on when you enter the study you may be asked to attend for a fifth visit*** (lasting approximately 1 hour). This will be a follow-up assessment and will take place 3 months from the date when you entered the study. **This visit will be a repeat of the first and fourth visits where you will complete the questionnaires, treadmill test and blood sample (optional).** You will be asked to wear a small, inconspicuous physical activity monitor on your thigh for the seven days following this visit.

The final visit (lasting 1 hour) will take place after the final assessment (usually within 4 weeks). You will be asked to attend a focus group with other participants in the research project. At the focus group you will be asked to talk about the study, and share your views on the TENS and the education session. The focus group will be recorded.

You will be reimbursed for travel expenses for these hospital visits.

## Where will the research be conducted?

## The research will be conducted in the Clinical Research Facility at the Queen Elizabeth University Hospital, Glasgow.

**What happens to the information?**

The information obtained will remain confidential and stored securely at the Robertson Centre for Biostatistics University of Glasgow. The data are held in accordance with the Data Protection Act, which means that we keep it safely and cannot reveal it to other people, without your permission. The data held on the database will not be identifiable. In addition, physical activity data and basic demographic data will be kept on a password protected database on a secure server at Glasgow Caledonian University. The data held on the database will not be identifiable. This information collected may be used for further analysis by staff and students in the School of Health & Life Sciences at Glasgow Caledonian University at a later date. If you are not eligible to continue the study after the second visit, we will keep the data collected up to that point and analyse it as part of the study.

**What are the possible benefits of taking part?**

These interventions are not routinely available in the NHS for your condition and their benefits have not been established. Therefore you might not benefit from taking part in the intervention. It is hoped that by taking part in this research, you will be providing valuable information regarding whether the use of the TENS machine and education may be beneficial in patients with PAD.

**What are the risks of the test involved?**

The risks in participating in this research are small. You will experience pain during the treadmill testing as you would normally experience on exercising. When you develop pain, it is expected to develop slowly and once you stop, the pain will resolve. You will be connected to a heart rate monitor throughout the test. There is a very unlikely chance that you will develop mild skin reaction to the activity monitor, or the TENS electrodes.

**What are my rights?**

We will inform you of the results of the study. We will contact your GP to let them know about your participation in the study. Participation in this study is entirely voluntary and you are free to refuse to take part or to withdraw from the study at any time without having to give a reason. Withdrawing from the study will not affect your future medical care or your relationship with medical staff looking after you.

**Will the research influence the treatment I receive?**

The research does not alter the treatment you receive. Your consultant and GP will start and stop treatments as determined by your clinical condition.

**Should I let my health insurance company know?**

Some insurance companies consider that participation in medical research such as this is a “material fact” which should be mentioned in any proposal for health-related insurance, or which could influence their judgment in consideration of claims made under existing insurance policies. You should check that participation in this research does not affect any policy you might be thinking about taking.

**Will my taking part in the study be kept confidential?**

The information collected about you in this study will be anonymised i.e. linked to a special code that is stored separately on a password-protected computer file. All information that is collected about you during the course of the research will be kept strictly confidential.

No one outside the research team will have any access to any identifying information. All identifiable information will be kept securely and will be retained for a minimum period of 5 years after the study ends.

**Who is organizing and coordinating the study?**

This study is being coordinated by Glasgow Caledonian University. The West of Scotland Research Ethics Committee that has responsibility for scrutinizing proposals for medical research on humans in Greater Glasgow and Clyde has examined the proposal and has raised no objections from the point of view of medical ethics. The study has also received approval from NHS GGC to proceed. It is a requirement that your records in this research, together with any relevant medical records, be made available for scrutiny by monitors from NHS GG&C.

**If you have any further questions?**

If you would like any further information regarding this study you can contact the researcher or the medical staff involved in the study (Contact details below)

Dr Chris Seenan

Department of Psychology, Social Work and Allied Health Sciences, Glasgow Caledonian University Cowcaddens Road Glasgow G4 0BA

Tel: (0)141 331 8151 Email: [Chris.Seenan@gcu.ac.uk](mailto:Chris.Seenan@gcu.ac.uk)

Professor Julie Brittenden

West Glasgow Ambulatory Care Hospital/ NHS GG&C / University of Glasgow

Tel: 0141 2019328 Email: [Julie.Brittenden@ggc.scot.nhs.uk](mailto:Julie.Brittenden@ggc.scot.nhs.uk)

Even after you agree to take part, you are still free to withdraw at any time and without giving a reason. A decision to withdraw at any time, or a decision not to take part, will not affect the standard of care you receive.

**Thank you for taking the time to read this Information Sheet and considering taking part in the study.**

We will give you a copy of the information sheet and signed consent form to keep. If you would like more information about the study and wish to speak to someone not closely linked to the study, please contact:

Dr Stephen McSwiggan

School of Medicine University of Dundee, Nethergate, Dundee, DD1 4HN,

Tel: 01382 383233Email: [s.j.mcswiggan@dundee.ac.uk](mailto:s.j.mcswiggan@dundee.ac.uk)

**If you have a complaint about any aspect of the study?**

If you are unhappy about any aspect of the study and wish to make a complaint, please contact the researcher in the first instance but the normal NHS complaint mechanisms is also available to you.

*This study has been reviewed by a NHS Research Ethics Committee, which has responsibility for scrutinising proposals for medical research on humans. In this case, the reviewing Committee was the West of Scotland Research Ethics Committee who have raised no objections from the point of view of medical ethics.’*
