# Supplementary material for: Efficacy and Feasibility of Pain management and Patient Education for Physical Activity in Intermittent claudication (PrEPAID): protocol for a randomised controlled trial
Source: Trials. 2019 Apr 16;20:222. doi: 10.1186/s13063-019-3307-6 (PMC6469131; doi:10.1186/s13063-019-3307-6)
Supplement: Supplementary file 3 — Participant consent form for PrEPAID trial. (DOCX 217 kb) [file 13063_2019_3307_MOESM3_ESM.docx]

**Addition files 3: PARTICIPANT CONSENT FORM**

**Title of Project:**

Pain Management and Patient Education for Physical Activity in Intermittent Claudication (PrEPAID): Feasibility Randomised Controlled Trial

**Name of Researchers:**

Dr Chris Seenan, Prof Julie Brittenden, Mr Abaraogu Ukachukwu, Dr Philippa Dall, Mr Wesley Stuart, Dr Garry Tew, Prof Jon Godwin

**Please initial the box if you agree with the following statements:**

1. I confirm that I have read and understand the information sheet Version 3.0 dated 12/09/207 for the above study and have had the opportunity to consider the information, ask questions and have had these answered satisfactorily.
2. I understand that participating in this study is voluntary and that I am free to withdraw at any point in time without giving reason and without my legal rights being affected.
3. I understand that relevant sections of my medical notes and data collected during the study may be looked at by individuals from collaborating Universities, or from the NHS GGC, where it is relevant to my taking part in this research. I give permission for these individuals to have access to my records
4. I consent to allow my data to be stored by the Robertson **Centre for Biostatistics**, and designated research related password protected computer for the purpose of this research and related future research.
5. I consent to allow my physical activity data and basic demographic data to be kept on a password protected database on a secure server Glasgow Caledonian University (GCU). The data held on the database will not be identifiable. This data may be used for further analysis and in comparison with future data, by staff and students of the School of Health & Life Sciences at GCU
6. I agree to my blood sample being taken as part of data collection for this study (optional), and to my blood sample being used in future ethically approved research
7. I agree to take part in the above study and understand that I may not continue to take part if deemed ineligible after the 2nd treadmill test

|  |  |  |  |  |
| --- | --- | --- | --- | --- |
| Name of Participant |  | Signature |  | Date |
|  |  |  |  |  |
| Name of Person taking consent |  | Signature |  | Date |
